# *In vitro* effects of tannin and extracts of *Bridelia ferruginea* and *Mitragyna inermis* on the exsheathment of infective larvae of *Haemonchuscontortus*

**DOI:** 10.1080/23144599.2020.1837056

**Published:** 2020-11-23

**Authors:** Esaïe Tchetan, Erick V. B. Azando, Pascal A. Olounladé, Géorcelin G. Alowanou, Sylvie M. Hounzangbé-Adoté

**Affiliations:** a Laboratoire d’Ethnopharmacologie et de Santé Animale, Faculté des Sciences Agronomiques, Université d’Abomey Calavi, Cotonou, Bénin; b Laboratoire de Biotechnologie et d’Amélioration Animale, Faculté des Sciences Agronomiques, Institut des Sciences Biomédicales Appliquées, Université d’Abomey-Calavi, Cotonou, Bénin; c Laboratoire d’Ecologie, de Santé et de Productions Animales, Département des Sciences et Techniques de Production Animale et Halieutique, Faculté d’Agronomie, Université de Parakou, Parakou, Bénin; d Laboratoire des Sciences Animale et Halieutique (Lasah), Unité de Recherches Zootechniques et Système d’Elevage, Ecole de Gestion et d’Exploitation des Systèmes d’Elevage, Université Nationale d’Agriculture, Porto Novo, Bénin; e Département des Sciences et Techniques Agricoles, Ecole Normale Supérieure de l’Enseignement Technique, Université Nationale des Sciences, Technologies, Ingénierie et Mathématiques, Abomey, Bénin

**Keywords:** Gastrointestinal nematodes, *B. ferruginea*, *M. inermis*, tannins, PVPP, *H. contortus*

## Abstract

*Bridelia ferruginea* (Euphorbiaceae) and *Mitragyna inermis* (Rubiaceae) are two plants of the beninese pharmacopeia used *in vivo* for the control of gastrointestinal nematodes (GINs) in small ruminants. The objective of the present study is to explore the mechanism of bioactive compounds involved in the action of these two plants on the third-stage infective larvae (L3s) of *Haemonchus contortus*. Thus, sheathed L3s of *H. contortus* were incubated with acetone extracts of *B. ferruginea* and *M. inermis* at concentrations of 0, 150, 300, 600 and 1200 µg/mL for 3 h at 25°C. The L3s were then washed and artificially submitted to exsheathment in the presence of sodium hypochlorite solution. The role of tannins was verified by adding a tannin inhibitor, polyvinyl polypyrrolidone (PVPP), to the acetone extracts of these two plants for 2 h at 25°C. Acetone extracts from *B. ferruginea* and *M. inermis* inhibited the exsheathment of *H. contortus* larvae (*p* < 0.001) and this inhibitory effect was dose-dependent for *M. inermis* at the concentrations tested. Treatment of *B. ferruginea* and *M. inermis* extracts with PVPP was associated with a partial restoration of the exsheathment kinetics of *H. contortus* larvae (*p* < 0.001), confirming the predominant role of tannins but also the residual role of other secondary metabolites. These *in vitro* results suggest that these plants are endowed with anthelmintic (AHs) properties and therefore likely to be used as alternatives to synthetic molecules.

## GRAPHICAL ABSTRACT

**Figure:**
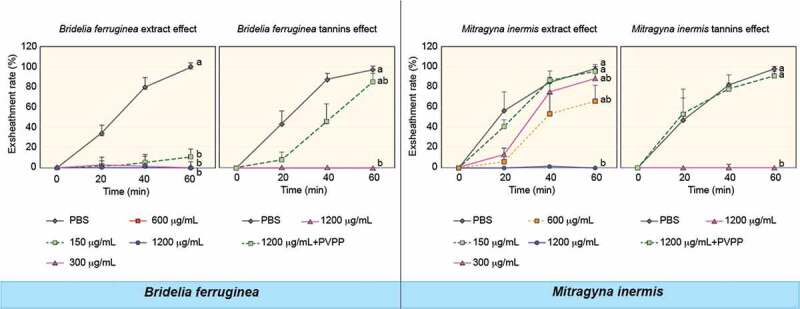

## 1. Introduction

Parasitic diseases caused by gastrointestinal nematodes (GINs) represent a serious pathological threat worldwide associated with the production of different species of grazing livestock, especially ruminants [1]. The principal way of controlling these parasites has been for long time through the use of chemical anthelmintics (AHs) molecules [2,3], which right now have shown their limits in several aspects.

In developing countries including the Benin republic, the synthetic AHs are often expensive and are not always available in both quantity and quality [4]. In addition, the development of resistance to synthetic AHs is now widespread throughout the pest population and has become a serious problem in some parts of the world [2,3]. Accessibility and counterfeits [5], residues and consumer expectations that are increasingly oriented towards farmed products with less chemical input are among the factors that limit the use of synthetic AHs.

This has led to the search for new and effective methods of pest management. Modern pest management therefore involves the search for alternative approaches combining the three principles of: (i) grazing management, (ii) stimulation of the host response and (iii) modulation of the parasite’s biology [6]. These approaches include the use of bioactive plants for their AHs properties. It is an option to improve the control of GINs and thus avoid pathological and physiological consequences on the host. Herbal remedies are an alternative in primary care systems and therefore a promising avenue for the development of improved traditional medicines [7].

Several ethnobotanical, ethnopharmacological and toxicological studies have been carried out in the world and especially in Africa in recent decades to identify and determine the mechanism of action of certain plants [8–10]. This is the case, for example, of studies on: *Carica papaya* [4]; *Newbouldia laevis* and *Zanthoxylum zanthoxyloides* [11–13]; *Onobrychis viciifoliae* [14]; *Anogeissus leiocarpus* and *Daniellia oliveri* [15] and *Crassocephalum crepidioides* [16].

Like the plants mentioned above, *Bridelia ferruginea* and *Mitragyna inermis* are two tropical shrubs widely found in West and Central African countries [17]. They have long been identified and used by local populations to treat various diseases in both humans and animals [10]. In humans, *B. ferruginea* and *M. inermis* have often been used to treat diabetes, malaria, dysentery and in animals, trypanosomiasis, diarrhoea, helminthosis [18–22]. Moreover, previous work has shown that both plants are not toxic [23–25].

Scientific work has been carried out on *B. ferruginea* and *M. inermis* on phytochemical screening [19,26–30] and the evaluation of the AHs properties attributed to them by the traditional pharmacopeia [10,31]. However, the mechanism of action of these plants on the GINs of small ruminants and the biological activity of secondary metabolites are not yet well known both *in vivo* and *in vitro*.

The present study therefore proposes to explore the nature of the bioactive compounds of these two plants on the third-stage infective larvae of *H. contortus*.

## 2. Materials and methods

### 2.1. Ethical approval

The present study was approved and conducted in accordance with the guidelines of the Ethical Committee of University of Abomey-Calavi (EC approval 2015/1134).

### 2.2. Plant identification and harvesting

The leaves of *B. ferruginea* (Euphorbiaceae) and *M. inermis* (Rubiaceae) were collected, identified and authenticated at the National Herbarium of the University of Abomey-Calavi under the numbers: AA6527/HNB and AA6529/HNB, respectively. The leaves of the mature plants collected in the municipality of Abomey-Calavi were dried in the laboratory at a temperature of 25°C. After 2 weeks of drying, they were transformed into powder using a laboratory grinder and kept at room temperature until use.

### 2.3. Extraction procedure

Extraction procedure and the yield of extracts were previously described [30]. Briefly, 50 grams (50 g) of powder from the leaves of each plant (*B. ferruginea* and *M. inermis*) were weighed and mixed in 500 mL of acetone and distilled water in a 70:30 ratio of distilled water-acetone. Extractions were made by maceration of the plant material. The mixture was magnetically stirred for 2 h at 50°C to break the molecular bonds and release the active substances. After filtration of the mixture, the filtrate was collected and evaporated under vacuum using a rotary evaporator. The extracts obtained were stored at 4°C in fridge.

### 2.4. *Obtaining L3s of* H. contortus

Third-stage infective larvae (L3s) were obtained following procedure described previously [30]. Briefly, faeces from sheep previously artificially infested with pure strains of *H. contortus* were collected and left in culture at room temperature for 10 days. The larvae were then extracted from the faecal mass by the Baermann apparatus. They were kept in the refrigerator at 4°C for at least 3 months.

### 2.5. Larval artificial exsheathment assay

The test was conducted according to the process described by Bahuaud et al. [32]. Sheathed L3s of *H. contortus* (2000 L3s/mL) were incubated for 3 h at 25°C with the extracts from each plant at concentrations of 0, 150, 300, 600 and 1200 µg/mL in phosphate-buffered saline (PBS). The L3s were then washed and centrifuged (67 × g) 3 times with PBS (pH 7.2). They were submitted to an artificial exsheathment process by adding an equal volume of 50 µL of a sodium hypochlorite solution containing 2.4% active chlorine previously diluted in the PBS at 1/75. Observation under a microscope (X 200) every 20 min after adding the sodium hypochlorite solution (0, 20, 40 and 60 min) and counting the L3s that began exsheathment process in relation to the total number of L3s made it possible to determine the kinetic of exsheathment. Five replications were made per plant extract for each concentration.

### 2.6. Highlighting the effect of tannins

To verify the role of tannins in larval exsheathment process, extract from each plant at concentration of 1200 µg/mL were placed in contact for 2 h at a temperature of 25°C in a ratio of 1:50 with polyvinyl polypyrrolidone (PVPP), which has the property of capturing tannin molecules, thus blocking their action [33]. The solutions were then centrifuged (1,358 × g) and the supernatant was removed and used for L3s incubation. Thereafter, the larval artificial exsheathment assay was performed according to the procedure described previously.

### 2.7. Statistical analyses

Exsheathment rate for each treatment was calculated by the following formula:

$$Td=\frac{Ld}{Ld+Le}\times 100$$

Td: Exsheathment rate; *Ld*: Number of L3s that began exsheathment process and *Le*: Number of L3s sheathed.

The recorded data was entered into the Excel® 2010 spreadsheet, which was used to calculate averages, standard deviations of the exsheathment rate and to generate the illustrative graphs. The data were subjected to non-parametric (Kruskal-Wallis) testing with R software (Version 2.15.3; 2013) to compare the averages of the exsheathment rate of the different treatments. The SNK test of NEWMAN and KEULS was applied for the structuring of the averages. Differences were considered significant at the 0.05% threshold.

## 3. Results

### 3.1. *Effect of extracts of* B. ferruginea *and* M. inermis *on L3s of* H. contortus

**Figure 1.**
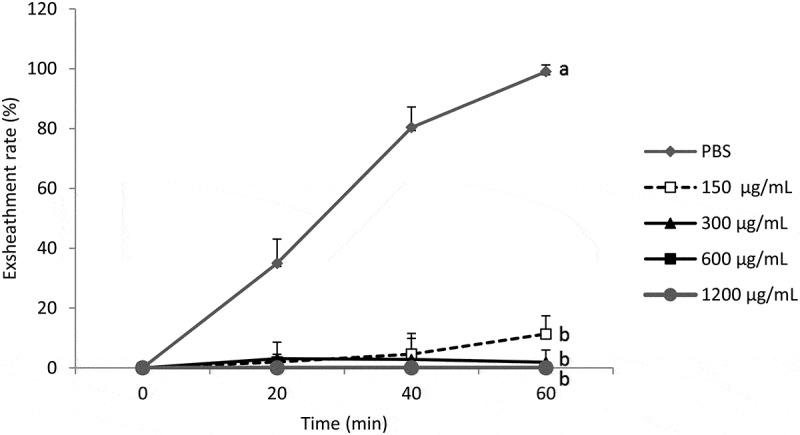
Effect of the acetone extract of *B. ferruginea* on the exsheathment kinetics of *H. contortus* larvae. Each curve represents the average exsheathment rate (as a function of time) for a given concentration ± Standard deviation, repetition = 5. The letters on each curve compare the results of different concentrations of acetone extract of *B. ferruginea*. Different letters indicate a significant difference in values at *p* < 0.05

Analysis of the results shows that the acetone extract of *B. ferruginea* affects the exsheathment kinetics of L3s of *H. contortus* (*p* < 0.001) and this inhibitory effect is not dose-dependent for the concentrations tested. The plant blocked the exsheathment of all larvae when used at concentrations of 300, 600 and 1200 µg/mL. Only at the concentration of 150 µg/mL allows to obtain exsheathment rate of 11.30% (Figure 1) of the larvae incubated with the extract of this plant after 60 min of contact.

**Figure 2.**
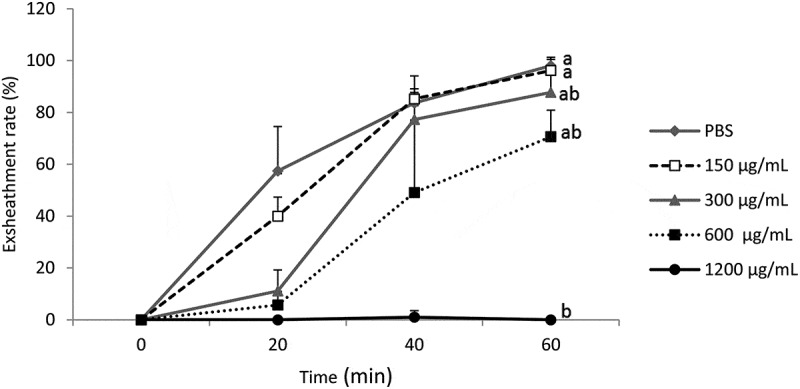
Effect of the acetone extract of *M. inermis* on the exsheathment kinetics of *H. contortus* larvae. Each curve represents the average exsheathment rate (as a function of time) for a given concentration ± Standard deviation, repetition = 5. The letters on each curve compare the results of different concentrations of acetone extract of *M. inermis*. Different letters indicate a significant difference in values at *p* < 0.05

On the other hand, the acetone extract of *M. inermis* affects the exsheathment kinetics of L3s of *H. contortus* (*p* < 0.001) with less efficiency than *B. ferruginea* and this inhibitory effect is dose-dependent for the concentrations tested. Apart from the concentration of 1200 µg/mL, which totally inhibited larval exsheathment, concentrations of 300 and 600 µg/mL disturbed larval exsheathment and the exsheathment rates obtained were 87.61% and 70.83%, respectively (Figure 2). The concentration of 150 µg/mL did not inhibit larval exsheathment.

With *B. ferruginea*, the structuring of the averages (Figure 1) shows that there is no significant difference between the exsheathment rate obtained with the different concentrations (150, 300, 600 and 1200 µg/ml) of *B. ferruginea* extract (*p* > 0.05). On the other hand, the difference between the negative control (PBS) and these different concentrations are highly significant (*p* < 0.001). This highlights the intensity of the inhibition of L3s exsheathment with *B. ferruginea* extract regardless of the concentrations tested.

*M. inermis* inhibited the exsheathment of L3s of *H. contortus* in a dose-dependent for the concentrations tested (Figure 2). The structuring of the mean of exsheathment rate showed that there was a significant difference between doses of 300 and 600 µg/mL and then 1200 µg/mL (*p* < 0.001). In contrast, the differences between PBS and concentrations of 150 then 300 and 600 µg/mL are not significant (*p* > 0.05).

### 3.2. Effect of tannins in the exsheathment disruption

**Figure 3.**
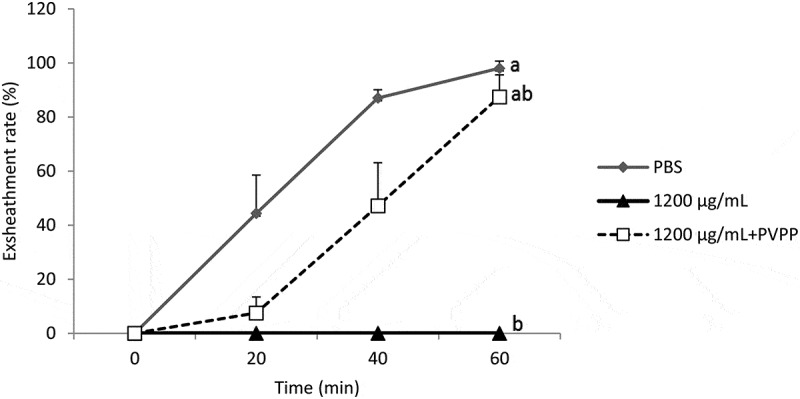
Demonstration of the effect of tannins in acetone extract of *B. ferruginea* on the kinetics of exsheathment of *H. contortus* larvae. Each curve represents the average exsheathment rate (as a function of time) for a given concentration ± Standard deviation, repetition = 5. The letters on each curve compare the results of different concentrations of acetone extract of *B. ferruginea*. Different letters indicate a significant difference in values at *p* < 0.05

**Figure 4.**
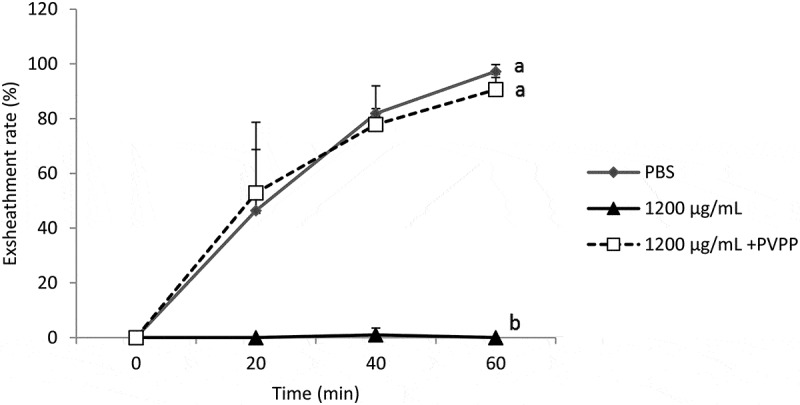
Demonstration of the effect of the tannins of the acetone extract of *M. inermis* on the exsheathment kinetics of the L3s of *H. contortus.* Each curve represents the average exsheathment rate (as a function of time) for a given concentration ± Standard deviation, repetition = 5. The letters on each curve compare the results of different concentrations of acetone extract of *M. inermis*. Different letters indicate a significant difference in values at *p* < 0.05

Treatment of *B. ferruginea* extract with PVPP was associated with an almost complete (87.94%) restoration of the exsheathment kinetics of *H. contortus* larvae (*p* < 0.001) (Figure 3), confirming the predominant role of tannins but also the partial role of other secondary metabolites. Similarly, treatment of *M. inermis* extract with PVPP was associated with an almost complete (90.96%) restoration of the exsheathment kinetics of *H. contortus* larvae (*p* < 0.001) (Figure 4). This shows the efficiency of the polyphenols of this plant on exsheathment kinetics disruption but also the inefficiency of other secondary metabolites.

The structuring of the average of exsheathment rate shows that there is no significant difference between the negative control (PBS) and the extracts incubated with PVPP (1200 µg/mL). On the other hand, the difference is significant between extracts treated with PVPP and those that were not treated (*p* < 0.001) (Figures 3 and 4), thus highlighting the share and efficiency of tannins from both plants in the disruption of the exsheathment kinetics of *H. contortus* larvae. This efficiency is more pronounced for *M. inermis* with exsheathment rate estimated at 90.96% at 60 min using the extract treated with PVPP, compared to 87.94% for *B. ferruginea*.

## 4. Discussion

Larval exsheathment is a physiological process that occurs when L3s are ingested by ruminants on pasture. It is an essential step in the continuation of larval migration in the internal phase of the life cycle of gastrointestinal nematodes (GINs). Inhibition of this exsheathment constitutes a blockage or even a break in the evolution of the worms’ life cycle. Thus, any plant whose consumption by small ruminants is associated with the inhibition of larval exsheathment has AH activity, particularly on the disturbance of the L3s installation. The results showed that extracts of *B. ferruginea* and *M. inermis* significantly affect (*p* < 0.001) the kinetics of exsheathment of *H. contortus* larvae and that at the tested doses (150, 300, 600 and 1200 μg/mL), this inhibitory effect depends on the concentration of extract for *M. inermis* and is independent of the dose for *B. ferruginea* for the concentrations tested. The intensity of inhibition is greater with *B. ferruginea* than with *M. inermis* with total inhibition of exsheathment already at 150 µg/mL. Similar results had been obtained on larval exsheathment inhibition by Azando et al. [11] with *Z. zanthoxyloides* and *N. laevis* on *H. contortus* and *T. colubriformis*, two other proven AHs plants in the beninese pharmacopeia for which tannins were held responsible for the AH property. In addition, *B. ferruginea* and *M. inermis* had also demonstrated AHs properties on the inhibition of larval migration of *H. contortus* [27] as had *Z. zanthoxyloides* and *N. laevis* [12].

Treatment of *B. ferruginea* and *M. inermis* extracts with PVPP was associated with partial restoration of L3s exsheathment kinetics, indicating that tannins play a major role in this inhibition where other secondary metabolites are also involved. The proportion of tannins is not identical for the two plants. Indeed, the efficiency of the inhibitory property is only 30% at 600 µg/mL for *M. inermis* while it is 90.96% the work of the tannins, while already at 150 µg/mL the inhibition is almost total with *B. ferruginea* for a tannin share of about 87.94%. Plants owe their properties to the richness of their chemical composition, which varies enormously according to several factors.

Phytochemical analysis of the two plants [19,26,27] revealed to varying degrees the presence of polyphenols, sterols, polyterpenes, flavonoids, quinonic compounds, saponosides, alkaloids, glycosides and carbohydrates.

Numerous other applied studies have demonstrated the effect of tannin in the control of GIN at all stages of development, from the inhibition of larval exsheathment [11,32,34–36], inhibition of larval migration of L3s [12,37,38], inhibition of egg hatch [30], inhibition of adult worm motility [11,30] or structural lesions on larvae or adult worms [39,40]. In this anthelmintic response, the hypothesis of a direct mode of action of the tannins has often been evoked but also the improvement of the host immune response [41].

## 5. Conclusion

The main objective of the present study was to evaluate the AH activity of *B. ferruginea* and *M. inermis* extracts on *H. contortus* larvae using a Larval Artificial Exsheathment Assay.

In summary, this experiment revealed that acetone extracts of *B. ferruginea* and *M. inermis* significantly inhibited the exsheathment of *H. contortus* larvae. Treatment of *B. ferruginea* and *M. inermis* extracts with PVPP was associated with an almost complete restoration of the exsheathment kinetics of *H. contortus* larvae, thus attesting to the predominant role of tannins from these two plants, especially for *M. inermis*, but also the partial role of other secondary metabolites. These results therefore lead to the conclusion that *B. ferruginea* and *M. inermis* can be used in the control of GINs of small ruminants especially in tropical areas where *H. contortus* has a high prevalence. However, further investigation is needed to better understand the mechanism of action of these plants on the entire internal phase of development of gastro-intestinal nematodes GINs and to isolate the molecules responsible for their AHs properties.
